# Feasibility and patient acceptability of a novel artificial intelligence-based screening model for diabetic retinopathy at endocrinology outpatient services: a pilot study

**DOI:** 10.1038/s41598-018-22612-2

**Published:** 2018-03-12

**Authors:** Stuart Keel, Pei Ying Lee, Jane Scheetz, Zhixi Li, Mark A. Kotowicz, Richard J. MacIsaac, Mingguang He

**Affiliations:** 1 Centre for Eye Research Australia, Royal Victorian Eye & Ear Hospital, Melbourne, Australia; 2 State Key Laboratory of Ophthalmology, Zhongshan Ophthalmic Center, Sun Yat-sen University, Guangzhou, China; 3 Department of Endocrinology & Diabetes, Barwon Health, Geelong, Australia; 4 Deakin University, Geelong, Australia; 5 Melbourne Medical School – Western Campus, Department of Medicine, The University of Melbourne, St Albans, Australia; 6 Department of Endocrinology & Diabetes, St Vincent’s Hospital, Melbourne, Australia; 7 Department of Medicine, The University of Melbourne, Parkville, Australia

## Abstract

The purpose of this study is to evaluate the feasibility and patient acceptability of a novel artificial intelligence (AI)-based diabetic retinopathy (DR) screening model within endocrinology outpatient settings. Adults with diabetes were recruited from two urban endocrinology outpatient clinics and single-field, non-mydriatic fundus photographs were taken and graded for referable DR ( ≥ pre-proliferative DR). Each participant underwent; (1) automated screening model; where a deep learning algorithm (DLA) provided real-time reporting of results; and (2) manual model where retinal images were transferred to a retinal grading centre and manual grading outcomes were distributed to the patient within 2 weeks of assessment. Participants completed a questionnaire on the day of examination and 1-month following assessment to determine overall satisfaction and the preferred model of care. In total, 96 participants were screened for DR and the mean assessment time for automated screening was 6.9 minutes. Ninety-six percent of participants reported that they were either satisfied or very satisfied with the automated screening model and 78% reported that they preferred the automated model over manual. The sensitivity and specificity of the DLA for correct referral was 92.3% and 93.7%, respectively. AI-based DR screening in endocrinology outpatient settings appears to be feasible and well accepted by patients.

## Introduction

Diabetic retinopathy (DR), the most common microvascular complication of diabetes, is a leading cause of irreversible vision loss in Australian adults of working age^1^. With the prevalence of diabetes predicted to increase substantially in the coming decades^2,3^, a significant increase in the health impact and economic burden of DR is expected in Australia^4^.

It is estimated that 98% of vision loss from DR is avoidable through early detection and treatment strategies^5,6^. As such, screening for DR has long been endorsed by the National Health and Medical Research Council (NHMRC), that recommend biennial screening for Australians without significant risk factors for retinopathy (e.g. poor glycaemic control, blood pressure or blood lipid controls) and annual screening (at least) for individuals who have had a diagnosis of DR^7^. Despite the growing evidence of the effectiveness of national screening programs worldwide^8,9^, comprehensive DR screening strategies have not been widely implemented in Australia, resulting in a suboptimal compliance with diabetic retinal examination guidelines^10^.

Medicare, a government funded health insurance system that provides free or subsidised health care services to the Australia population, has recently introduced a new initiative that supports non-eye care professionals to perform screening for DR using retinal photography^11^. While retinal photography is an effective screening tool for DR, it is highly dependent on interpretation by clinical experts^12^, posing a significant challenge to this model. Over the past two decades, automated techniques for the assessment of DR, via electronic medical records^13,14^ and feature extraction from color retinal images^15,16^, have been reported with variable accuracy. The recent emergence of deep learning-based artificial intelligence grading of DR represents an advancement of artificial neural networks that permit improved accuracy of disease classification (sensitivity and specificity > 90%) from raw image data^17,18^. These systems offer the potential to enable non-eye health professionals to perform opportunistic screening for DR and to make referral recommendations without the need for eye specialists. Despite this, there is a paucity of data relating to the clinical adoption of these technologies.

In light of this, we propose a novel and innovative screening strategy, combining an AI-based automatic grading algorithm for the detection of DR with the accessibility of endocrinology clinics. The purpose of this pilot study was to evaluate the feasibility and patient acceptability of this opportunistic screening model in two public hospital endocrinology outpatient clinics.

## Methods

### Participants

Patients who attended two urban endocrinology departments (St Vincent’s Hospital, Melbourne & University Hospital Geelong, Barwon Health) for diabetes care between July 6^th^, 2017 and November 2^nd^, 2017 (equivalent of 13 clinic days) were invited to participate in this study if they were at least 18 years of age. For all eligible persons, a response as to whether participants accepted or refused participation was recorded after clearly explaining the project objectives and rationale for the AI-based automated retinal screening. For those who declined, a reason for refusal was obtained. At each screening site, a convenience sample of 50 eligible patients were recruited. Ethics approval was obtained from the St Vincent’s Hospital and Barwon Health (HREC-LNR/17/SVHM/39) Human Research Ethics Committee’s. Study procedures adhered to the tenets of the Declaration of Helsinki as revised in 2013 and participants provided written informed consent to participate.

### Development of a deep learning algorithm for referrable DR

The deep learning algorithm was developed using 66,790 retinal photographs acquired from an online dataset (http://www.labelme.org, Guangzhou, China). Using a simple random sampling method, a total of 58,790 images were assigned to the training dataset and the remaining 8,000 images were held-out for internal cross-validation. Amongst the 58,790 images in the training dataset, 10,861 (18.5%) had referable DR and 13,736 (27.5%) had diabetic macular edema (DME). In the development of this algorithm, 21 ophthalmologists, who achieved robust agreement (Kappa ≥ 0.70) with an experienced ophthalmologist on a test set of 180 retinal images, were recruited as graders. Retinal images were graded according to the NHS diabetic eye screening guidelines that have been described in detail elsewhere^19^. Referable DR was defined as moderate non-proliferative DR (NPDR) or worse and/or diabetic macular edema (DME). Each image was randomly assigned to a single ophthalmologist for initial grading and, following this, sequentially assigned to individual graders until three consistent grading outcomes were achieved. The consensus grading outcome was assigned as the final, conclusive grading of each image.

The system included four deep learning models, all using inception-v3 architecture training from scratch with a mini-batch gradient descent size of 32 and Adam optimizer of a 0.002 learning rate^20^. This included networks for the (1) classification for referable DR, (2) classification of DME, (3) evaluation of image quality for DR, and (4) assessment of image quality and of the availability of macular region for DME. The model used in this study was version 20170520.

### Protocol

**Figure 1:**
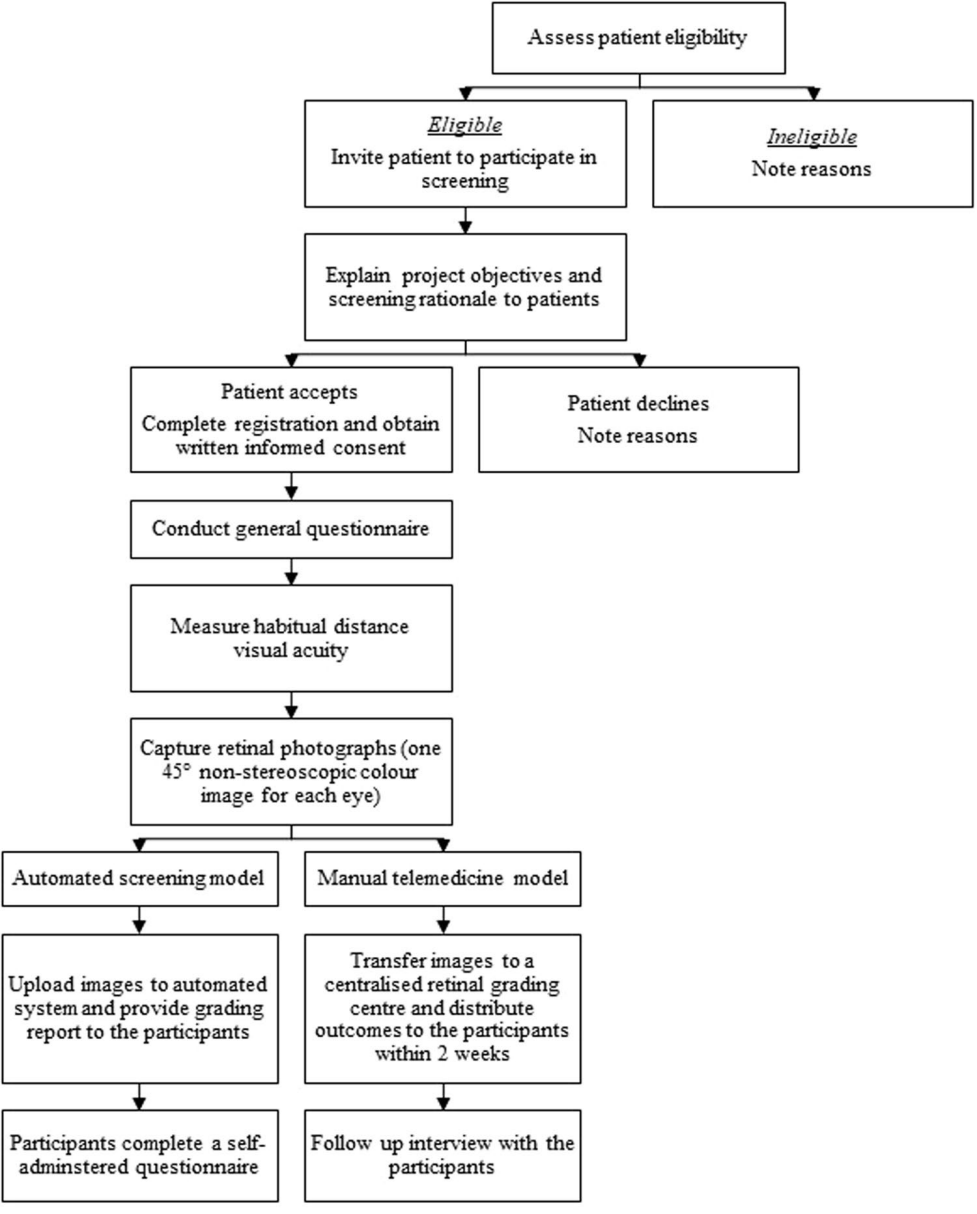
Flowchart of testing protocol.

All eligible and consenting participants underwent a general questionnaire to collect information on sociodemographic factors, previous ocular history, general health and use of eye health care services. Presenting distance visual acuity was measured in each eye using a logMAR chart (Brien Holden Vision Institute, Australia) in well-lit room conditions. Participants wore their habitual distance correction. One standard, 45-degree, non-stereoscopic colour retinal photograph (central-nasal field) was taken of each eye using a Digital Retinography System (DRS, CenterVue SpA, Italy). Each participant underwent; (1) automated screening model, where retinal photographs were uploaded to EyeGrader^TM^ (Healgoo Interactive Medical Technology Co. Ltd, Guangzhou, China), an online platform that enables retinal photograph upload, automated grading and generation of a single-page grading report including referral recommendations that was delivered to the patient at the time of consultation; and (2) manual telemedicine model, where retinal images were transferred to a centralised retinal grading centre and manual grading outcomes were distributed to the patient within 2 weeks of baseline assessment. Figure 1 shows a flowchart of the testing protocol.

On the day of examination, participants completed a self-administered modified client satisfaction questionnaire (CSQ-8) to collect details on their overall satisfaction with the automated model of care and likelihood of using the screening service again. Participants were recontacted (via their preferred model) at 1-month following baseline assessment to confirm whether they had accessed their manual grading report and determine their preferred model of care. If participants could not be contacted after 3 attempts, they were not interviewed. The impact of the automated screening model on clinic flow was assessed using the average time taken for each screening episode per participant. Participants were provided with a referral to an optometry service if (a) their presenting visual acuity was <6/12 (current Australian driving standard) in either eye; or (b) they had referable DR.

### Statistical analysis

We calculated descriptive statistics of the characteristics of patients and the average time taken for the automated DR screening model. Continuous variables were presented as mean (SD) and categorical variables were presented as absolute (n) and relative frequencies (%). The performance of the deep learning algorithm was evaluated using the ophthalmologist assessment as the reference standard. Sensitivity and specificity were the key outcome measures. All statistical analysis was performed using Stata version 11 software (College Station, Texas, USA).

## Results

### Feasibility and acceptability of automated DR screening

Over the 4-month study period, a total of 128 participants were invited to participate, of whom, 96 (75%) agreed and were subsequently screened for DR. The reasons for which individuals declined to participate included that they had a recent eye test (16/32, 50%), they had an upcoming appointment with an eye-care professional (7/32, 22%), or that they were not interested (3/32, 9%). The remaining 6 (6/32, 19%) individuals did not disclose a reason for refusal. The mean age of the 96 study participants was 44.26 +/− 16.56 years (range = 20–90 years), 57% (55/96) were male and the mean duration of diabetes was 18.2 +/− 13.5 years. Twenty-five percent (24/96) of participants indicated that they had not had an eye examination for diabetes in the preceding 12 months.

Of the total 96 participants, 93 (96.9%) had a retinal photograph in at least one eye that was gradable for referrable DR (gradable images in only one eye = 10.8%, 10/93) using the deep learning algorithm. Of the three participants with bilateral ungradable retinal photographs, two were a result of small pupil size and one was due to bilateral media opacities. In the manual screening model, a grade for referrable DR was obtained in at least one eye for all participants. The mean time for the automated screening model was 6.9 +/− 1.4 minutes. The sensitivity and specificity of the DLA for correct referral was 0.933, 92.3% and 93.7%, respectively. In total, 17 participants received a referral from the automated screening model. Of these, 12 were true positive referrals (referable DR = 10; VA < 6/12 = 2), 5 were false positives due to a misclassification of mild NPDR. There was one false negative case that proved to be undetected stable proliferate DR with subthreshold panretinal laser scars on manual grading.

Ninety-six percent (92/96) of participants reported that they were either satisfied or very satisfied with the automated screening model. None of the four participants who were unsatified with automated screening specified a reason. Eighty-nine percent (85/96) stated that they would be likely or extremely likely to use the service again and 89% (85/96) reported that they would recommend the automated retinal screening system to a friend.

### Participant follow-up

Only 57% (55/96) of participants were able to be contacted 1-month following baseline assessment after 3 attempts by researchers. Of these, 6 (11%) participants had not accessed their manual screening report and 78% (43/55) reported that they preferred the automated model over the manual model. A small number of participants (n = 5) commented that the reason for preferring automated grading was the convenience of immediate reporting of results. Of the 12 participants who preferred manual screening, only 50% (6/12) disclosed a reason. All 6 respondents stated that ‘trust’ was the key reason for preferring manual professional grading over machine diagnosis.

Among the 5 participants who were misclassified, only 2 responded to the telephone interview (after 3 attempts). Of these, one preferred the automated model and the other preferred the manual model. Due to the low response amongst these individuals at follow-up, we are unable to make any robust discussion on whether misclassification by the automated model impacted a participant’s decision to choose manual grading as their preferred screening method.

## Discussion

This pilot study indicates that our AI-based DR screening model appears to be feasible, accurate and well accepted by patients attending endocrinology outpatient settings. Herein, we describe novel data of the clinical adoption of a deep-learning algorithm for the detection of referable DR within two Australian endocrinology outpatient clinics.

Diabetic retinopathy screening strategies have long been trialled in non-ophthalmic settings in Australia with modest success^21,22^. Typically, these models integrate retinal photography and a centralised retinal grading centre that require support by highly trained professionals and involve a delay in communicating screening results to patients. Drawbacks of the manual DR screening model were highlighted in this study, with over 40% of patients unable to be re-contacted after 3 attempts and an additional 11% not accessing their manual grading report. The automated screening model, on the other hand, offers a technology-driven screening solution that enables point-of-care reporting of results, thereby potentially addressing many issues associated with the delayed communication of results including patient anxiety, documentation errors and difficulties re-contacting patients^23^.

Our finding that more than 10% of participants had referrable DR is higher than a recent population-based report^24^. In addition, one quarter of participants had not undertaken annual DR screening providing evidence that endocrinology outpatient clinics may offer an ideal setting to opportunistically capture individuals with referrable disease and those who do not routinely attend eye care services for routine examinations. The AI-based diagnostic assistive technology utilised in this study coupled with the recent introduction of Medicare items for non-eye health physicians to screen for DR^11^ offers the potential to build sustainable DR screening within endocrinology settings.

There are several findings from this pilot study that indicate that the AI-based DR screening in endocrinology outpatient settings appears to be feasible, including; (1) the software was successfully incorporated with a fully automated retinal camera that requires minimal operator training; (2) the mean time per automated screening episode was approximately seven minutes, fitting in well with the routine clinical workflow; and (3) only 3% of participants had ungradable retinal images as classified by the deep learning algorithm, providing evidence of reliable image analysis using non-mydriatic photography. Furthermore, the performance indicators (sensitivity and specificity) are substantially better than what screening guidelines would typically recommend (>80% sensitivity and specificity)^18^. Despite this, given the small number of referrable cases in the present study, further validation in larger patient cohorts is warranted.

A key challenge to the clinical adoption of this AI-based technology relates to a mind-set shift in how patients entrust clinical care to machines. In this study, 96% of participants reported that they were either satisfied or very satisfied with the automated screening model and nearly 80% reported that they preferred the automated screening model over the manual model, suggesting a good degree of patient acceptability of the system. Further research investigating clinician and organisational stakeholder acceptance, experiences and perspectives is required in order to assist in designing a sustainable service delivery model.

The key strength of this study includes its prospective study design that provides some of the first evidence of the adoption of a deep-learning algorithm in a point-of-care DR screening model. A number of limitations must also be considered. First, there was a relatively small representation of referrable cases in the sample, which may have resulted in an unstable estimate of diagnostic accuracy. It should also be noted that contrast between retinal background and DR lesions varies considerably among ethnicities, and is therefore a source of potential error for automatic detection systems. Although not detailed in this report, our algorithm has been validated in four ethnic groups, including Chinese (AUC = 0.989), Malay (AUC = 0.962), Caucasian (AUC = 0.969) and Indigenous Australian (AUC = 0.937), each with distinct retinal pigmentation (data not shown). Second, given the primary objectives of this study were to assess technical feasibility and patient acceptability, all patients aged 18 years and over were invited to participate in this pilot study. This does not accurately simulate the real-world application of this model, where only those who have not undertaken an annual retinal exam would be screened. Third, at first glance, the decline rate of 25% is noteworthy. However, 72% (23/38) of those who declined reported having had a recent eye examination or an upcoming eye-care appointment and were therefore unlikely to have benefited from this endocrinology-based DR screening. Lastly, we did not evaluate the economic effectiveness of the automated screening model, nor did we assess adherence to referral rates. We plan to address these limitations in a longer term, multi-centre study. Future research will also focus on adopting the automated screening model to general practitioner clinics. Given 85% of the Australian population visit this service at least once every 12 months^25^, they too can potentially serve as a venue for opportunistic screening of DR.

In summary, this AI-based DR model appears to be feasible and well accepted by patients attending two endocrinology outpatient settings. Further work to investigate the impact (i.e. adherence to referral, new DR detection rates) and cost effectiveness of this model is ongoing.

### Data Availability

The datasets generated during and/or analysed during the current study may be available from the corresponding author on reasonable request.
